# Evaluation of Retinal Structure and Visual Function in Blue Cone Monochromacy to Develop Clinical Endpoints for L-opsin Gene Therapy

**DOI:** 10.3390/ijms251910639

**Published:** 2024-10-02

**Authors:** Artur V. Cideciyan, Alejandro J. Roman, Raymond L. Warner, Alexander Sumaroka, Vivian Wu, Yu Y. Jiang, Malgorzata Swider, Alexandra V. Garafalo, Iryna Viarbitskaya, Robert C. Russell, Susanne Kohl, Bernd Wissinger, Caterina Ripamonti, John L. Barbur, Michael Bach, Joseph Carroll, Jessica I. W. Morgan, Tomas S. Aleman

**Affiliations:** 1 Scheie Eye Institute, Department of Ophthalmology, Perelman School of Medicine, University of Pennsylvania, Philadelphia, PA 19104, USA; aroman@pennmedicine.upenn.edu (A.J.R.); raymond.warner@pennmedicine.upenn.edu (R.L.W.); asumarok@pennmedicine.upenn.edu (A.S.); vivian.wu@pennmedicine.upenn.edu (V.W.); yuyou.jiang@pennmedicine.upenn.edu (Y.Y.J.); mswider@pennmedicine.upenn.edu (M.S.); garafalo@pennmedicine.upenn.edu (A.V.G.); iryna.viarbitskaya@pennmedicine.upenn.edu (I.V.); robert.russell2@pennmedicine.upenn.edu (R.C.R.); jwmorgan@pennmedicine.upenn.edu (J.I.W.M.); aleman@pennmedicine.upenn.edu (T.S.A.); 2 Molecular Genetics Laboratory, Centre for Ophthalmology, University of Tübingen, D-72076 Tübingen, Germany; susanne.kohl@med.uni-tuebingen.de (S.K.); bernd.wissinger@med.uni-tuebingen.de (B.W.); 3 Cambridge Research Systems, Rochester ME2 4BH, UK; c.ripamonti@crsltd.com; 4 Centre for Applied Vision Research, School of Health & Psychological Sciences, City St. George’s, University of London, London EC1V 0HB, UK; j.l.barbur@city.ac.uk; 5 Eye Center, Medical Center—Faculty of Medicine, University of Freiburg, D-79106 Freiburg, Germany; bach@uni-freiburg.de; 6 Departments of Ophthalmology & Visual Science, Cell Biology, Neurobiology, and Anatomy, Medical College of Wisconsin, Milwaukee, WI 53226, USA; jcarroll@mcw.edu

**Keywords:** color vision, microperimetry, outcome measures, perimetry

## Abstract

L-cone opsin expression by gene therapy is a promising treatment for blue cone monochromacy (BCM) caused by congenital lack of long- and middle-wavelength-sensitive (L/M) cone function. Eight patients with BCM and confirmed pathogenic variants at the OPN1LW/OPN1MW gene cluster participated. Optical coherence tomography (OCT), chromatic perimetry, chromatic microperimetry, chromatic visual acuity (VA), and chromaticity thresholds were performed with unmodified commercial equipment and/or methods available in the public domain. Adaptive optics scanning laser ophthalmoscope (AOSLO) imaging was performed in a subset of patients. Outer retinal changes were detectable by OCT with an age-related effect on the foveal disease stage. Rod and short-wavelength-sensitive (S) cone functions were relatively retained by perimetry, although likely impacted by age-related increases in the pre-retinal absorption of short-wavelength lights. The central macula showed a large loss of red sensitivity on dark-adapted microperimetry. Chromatic VAs with high-contrast red gratings on a blue background were not detectable. Color vision was severely deficient. AOSLO imaging showed reduced total cone density with majority of the population being non-waveguiding. This study developed and evaluated specialized outcomes that will be needed for the determination of efficacy and safety in human clinical trials. Dark-adapted microperimetry with a red stimulus sampling the central macula would be a key endpoint to evaluate the light sensitivity improvements. VA changes specific to L-opsin can be measured with red gratings on a bright blue background and should also be considered as outcome measures in future interventional trials.

## 1. Introduction

Blue cone monochromacy (BCM) is an X-linked inherited retinal disease (IRD) caused by pathogenic variants in the OPN1LW/OPN1MW gene cluster, encoding long- and middle-wavelength-sensitive (L/M) cone opsins [1,2]. Two of the most common mutations in the USA and Europe are deletions encompassing the locus control region (LCR) and parts of the OPN1LW/OPN1MW gene cluster or a C203R point mutation. Both types of mutations result in an identical phenotype with severe dysfunction of L/M cones, which are the major contributors to day vision in healthy eyes [3,4,5,6,7,8,9,10]. However, foveal structural changes tend to progress faster in patients with large deletions as compared with the point mutation [7]. All patients have serious day vision impairment consisting of reduced visual acuity, impaired color vision, nystagmus, and, in many cases, increased photosensitivity. Most BCM patients have healthy night vision mediated by functioning rod photoreceptors. Importantly, the daylight vision of BCM patients is mediated by the combination of short-wavelength-sensitive (S) cone photoreceptors and rod photoreceptors that function in a partially desensitized manner in a daylight environment [3,4,5,6,7,8,9,10]. There are currently no treatments for BCM, and management of the disease is limited to the use of tinted lenses and low vision aids.

The overarching goal of any BCM therapy is to express L/M opsin in L/M cone photoreceptors across the retina to improve day vision. This goal must be achieved at a stage of the disease when enough L/M cones are surviving (despite being dysfunctional) and receptive to therapy (such as retaining the molecular machinery required for phototransduction and synaptic signaling). Additionally, post-receptoral cells in the retina and in the visual brain need to be receptive to processing and interpreting a new source of visual input originating from the retina. The consequences of expressing an exogenous opsin in the retina have been previously evaluated in animal models with varied similarities to BCM, laying the foundation for translation to patients [11,12,13,14,15,16].

ADVM-062 was recently developed as a one-time intravitreal injection designed to express sustained levels of L-opsin in cones for treating BCM [17]. ADVM-062 is a recombinant single-stranded adeno-associated virus (AAV) construct, flanked by AAV2 inverted terminal repeat (ITR) sequences packaged in the AAV.7m8 capsid variant [18,19,20,21,22,23]. ADVM-062 has MNTC regulatory sequences that include the LCR and a minimal M-opsin promoter designed for preferential expression in L/M cones. When tested in Mongolian gerbils which have cone-rich retinas naturally lacking L-opsin, ADVM-062 showed in vivo pharmacological activity, with a significant increase in retinal sensitivity to red light, without affecting the function of the natural M- or S cones [17]. In addition, there is evidence of cone-specific expression in gerbils [17]. When tested in non-human primate eyes without disease, ADVM-062 was mostly expressed in foveal cones, consistent with the design features of an intravitreal injection [17]. Unexpectedly, however, a subset of parafoveal and peripheral cones of primates, including up to 30% of S cones, also expressed the exogenous L-opsin [17]. Thus, in BCM patients with the early stages of disease and with a retained foveal structure, ADVM-062 provides the potential to confer long-wavelength sensitivity to congenitally silent foveal and peripheral L/M cones. In addition, for BCM patients with foveal degeneration there may be a secondary pathway of augmenting long-wavelength sensitivity by bestowing expanded wavelength sensitivity to a subset of S cones. The aims of the current prospective study were to quantify and to localize spectrally distinct visual function at foveal and extrafoveal locations mediated by specific photoreceptor types, with the goal of developing a clinical protocol with outcome measures based on commercially or publicly available equipment.

## 2. Results

### 2.1. Range of Outer Retinal Disease Severity

**Figure 1:**
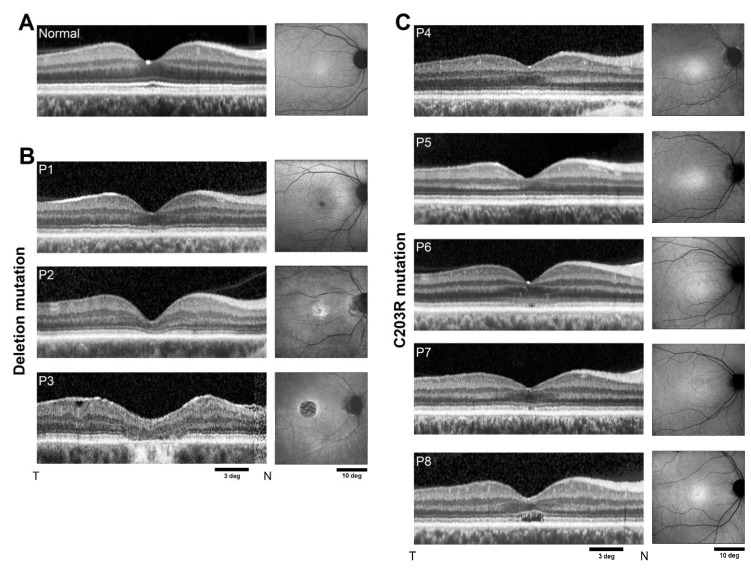
Range of disease severity in BCM patients. OCT along the horizontal meridian (left panels) and NIR-RAFI (right panels) in a representative normal (**A**) compared with patients with deletion mutations (**B**) and those with C203R mutations (**C**). Calibration bars are shown. T, temporal retina; N, nasal retina; OD, right eye; OS, left eye.

The range of disease severity represented in the current cohort of BCM patients was evaluated with optical coherence tomography (OCT) scans along the horizontal meridian crossing the fovea (Figure 1 and Supplementary Figure S1). Qualitatively, temporally averaged scans P4 and P5 at ages 11 and 19 years showed near-normal retinal lamination but were missing the layer originating from cone outer segment tips (COST, also known as the interdigitation zone) (Figure 1C). Near-infrared reduced-illuminance autofluorescence (NIR-RAFI), dominated by melanin-related pigments of the RPE, was near-normal with some minor heterogeneity visible in P5 (Figure 1C). Quantitatively, both P4 and P5 had reduced outer nuclear layer (ONL) thickness at the fovea (64 and 55 µm, normal lower limit 75.3 µm, N = 22, age range 8–62 years), and the layer originating near the junction between the inner and outer segments (IS/OS) was disrupted on spatially averaged scans (Supplementary Figure S1, arrows). Thus, both patients would be classified as presenting Stage 3 foveal disease [7]. P1, P6, and P7, at ages 21 to 24, showed patchy disruption of the IS/OS band on temporally averaged (Figure 1B,C) and spatially averaged (Supplementary Figure S1) OCT scans. NIR-RAFI was near normal in P6 but showed macular heterogeneity in P7 and a lack of normal central hyper-autofluorescence in P1 (Figure 1B,C). Quantitatively, all three patients showed reduced ONL thickness at the fovea (66, 71, and 65 µm). Thus, P1, P6 and P7 were also classified as presenting Stage 3 foveal disease. P2 and P8, at ages 46 and 30 years, respectively, showed major disruptions to the foveal IS/OS line with (P8) and without (P2) the appearance of an optical gap (Figure 1B,C). NIR-RAFI was near normal in P8, with heterogeneity visible in P2. Foveal ONL thickness was reduced (46 and 53 µm). Thus, P2 and P8 would be classified as having Stage 4 foveal disease. P3, at age 66, had an atrophic macula and Stage 5 foveal disease. The best corrected visual acuities (BCVA) ranged from 0.60 to 0.94 logMAR (Table 1) corresponding to Snellen equivalent range of 20/80 to 20/160.

### 2.2. Extrafoveal Visual Function

**Figure 2:**
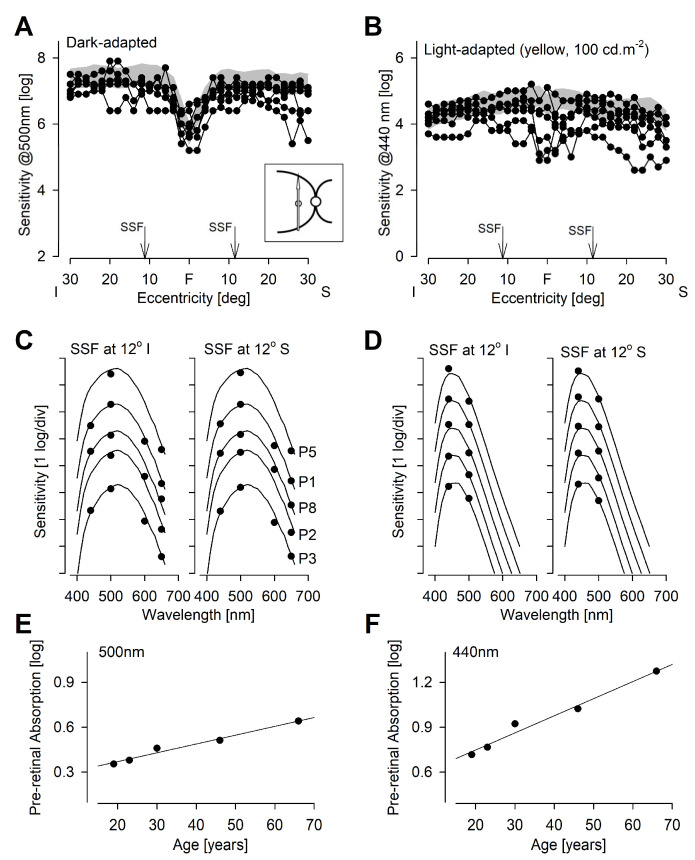
Rod and S cone sensitivity distribution. (**A**) Dark-adapted sensitivity to 500 nm stimuli along the vertical meridian in BCM patients (connected symbols) compared with normal limits (gray band). (**B**) S cone sensitivity profiles (connected symbols) of the BCM patients using a 440 nm stimulus on a yellow adapting background compared with normal limits (gray band). (**C**,**D**) Spectral sensitivity functions (SSF) in a subset of BCM patients measured at 12° eccentricity in superior and inferior fields under dark-adapted (**C**) and yellow-adapted (**D**) conditions. Results from each patient (symbols) shifted vertically for visibility, fit by rod (**C**) and S cone (**D**) photoreceptor sensitivities adjusted for pre-retinal absorption for each in-dividual, and ordered by age. (**E**,**F**) Individualized pre-retinal absorption estimates (symbols) at 500 nm (**E**) and 440 nm (**F**) obtained by best ensemble fit functions shown in panels (**C**) and (**D**), respectively. Regression lines shown.

To better understand the photoreceptor origins of the light sensitivity in BCM patients, we performed free-viewing chromatic perimetry along the vertical principal meridian under dark-adapted and light-adapted conditions (Figure 2). Perimetric data obtained from P4 appeared to outlying (likely due to the young age) and were censored from further consideration. Under dark-adapted conditions, sensitivity to 500 nm (blue-green) stimulus along the vertical meridian was within the normal range for four patients (P1, P5, P6, and P7) and showed minor losses of less than 1 log for the remaining patients (Figure 2A). Under yellow (100 cd.m^−2^) light-adapting conditions, sensitivity to a 440 nm (violet) stimulus along the vertical meridian were within the normal range for four patients (P1, P5, P6, and P8) and showed minor losses for the remaining patients (Figure 2B).

As two of the oldest patients (P2 and P3) showed greater losses of sensitivity as compared with younger patients, we examined the possible contribution of age-related lens yellowing [24]. Multi-spectral sensitivities recorded in a subset of five patients at two retinal locations under both dark-adapted and yellow-adapted conditions were fit as an ensemble with rod and S cone photoreceptor sensitivities adjusted for pre-retinal absorption for each individual (Figure 2C,D). The magnitude of the individualized adjustments for pre-retinal absorption showed a distinct linear correlation with age at 500 nm (Figure 2E) and at 440 nm (Figure 2F). Importantly, foveal disease stage was also correlated with mean rod sensitivity (R^2^ = 0.57) and mean S cone sensitivity (R^2^ = 0.61) implying the possible existence of multiple aging effects. Previous work has shown a complex mediation with longer wavelength stimuli driven by rod function and shorter wavelength stimuli driven by S cone function under standard white-light-adapting conditions [4].

### 2.3. Visual Function Immediately Surrounding the Anatomical Fovea

**Figure 3:**
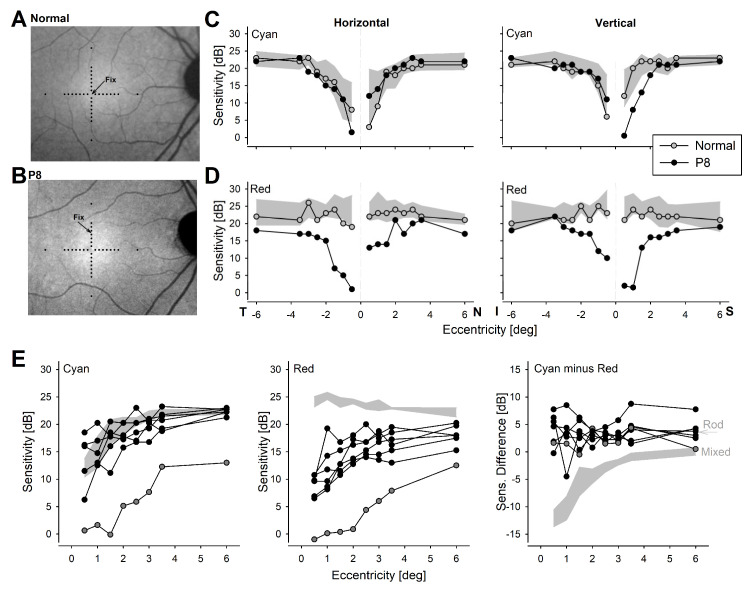
Chromatic sensitivities at the central retina. (**A**,**B**) NIR-RAFI in representative normal (**A**) and P8 (**B**). Retinal locations sampled (black symbols) and the center of fixation (Fix) are shown. (**C**,**D**) Microperimetric sensitivity profiles in the normal and P8 along the horizontal and vertical meridians with cyan (**C**) and red (**D**) stimuli. Gray region is the normal range. (**E**) Sensitivities of all BCM patients as a function of eccentricity to cyan (left panel) and red (middle panel) stimuli. Dark gray symbols are from the oldest subject, P3. Cyan minus red sensitivity difference is also shown (right panel). Gray regions show the normal ranges in each panel, the gray arrow indicates the difference expected from rod mediation in the extramacular region.

We used two-color retina-tracking perimetry (also called microperimetry) to evaluate the function at the para-/peri-foveal region using a custom cross-shaped test pattern (Figure 3). It is important to note that subjects were fully dark-adapted before each test but the test itself can only be performed with a dim red background and thus it is not fully dark-adapted. Results from P8 and a representative normal subject allow better understanding and interpretation of the results (Figure 3A–D). A normal subject had stable and foveal fixation (Figure 3A), whereas P8 had a variable fixation located at ~2° superior to the fovea (Figure 3B). Along the horizontal and vertical meridia, at the perifoveal 6° eccentricity loci and with cyan stimuli, both the normal subject and P8 had sensitivities of 22 dB each on average (Figure 3C). With red stimuli, average sensitivities at 6° eccentric were 21 dB and 18 dB for normal and P8, respectively (Figure 3D). Cyan minus red sensitivity differences were 4 dB and 1 dB for normal and P8, respectively. In the normal subject, though not in P8, an additional locus at the rod hotspot [25] (at 12° superior retina) produced 22.3 and 18.7 dB for cyan and red sensitivities, respectively, with a 3.6 dB difference. Assuming sensitivities were rod-mediated at the normal rod hotspot, these results suggest a slight intrusion of mixed mediation (i.e., cyan perceived by rods, red by cones) in the normal eye at 6° eccentricity. Knowing the BCM functional defect, parsimony suggests that red sensitivities in P8 at 6° eccentric are rod mediated. Cyan sensitivities in P8 are also likely to be rod mediated as normal dark-adapted S cone sensitivity to that stimulus is expected to be at the level of 5 dB or lower.

Cyan sensitivities of both P8 and the normal subject decreased from the perifoveal loci towards the fovea with a steeper roll-off occurring within 2–3° of the foveal center (Figure 3C). This roll-off was likely driven by a complex combination of increasing macular pigment density, reducing rod cell density, and shortening rod outer segments as the foveal center is approached from the perifovea. With red stimuli, however, the pattern between the normal subject and P8 were very different (Figure 3D). The normal subject showed increasing sensitivity towards the fovea, likely driven by the increasing numbers of L/M cones and lengths of their outer segments, whereas P8 showed reducing red sensitivity following the pattern of the cyan sensitivity roll-off. These results imply differences in photoreceptor mediation between the normal subject and the representative BCM patient.

Across all BCM patients, data were summarized as a function of eccentricity from the anatomical fovea averaged along two meridia and compared with the normal (gray bands) range (Figure 3E). Cyan sensitivities were at or near the normal range for all patients except for one (Figure 3E, left). Red sensitivities, however, were abnormal in all BCM eyes (Figure 3E, middle). The magnitude of the red sensitivity loss grew from 6° eccentric towards the fovea to reach nearly 2 log units near the fovea. In normal, cyan minus red chromatic differences were near 0 dB at 6° eccentric. However, as the fovea was approached, normal chromatic differences became substantially negative. In BCM patients, chromatic differences were invariant between fovea and 6° eccentric, and averaged +3.6 dB similar to the difference observed at the normal rod hotspot (Figure 3E, right). There was no obvious relationship between age and sensitivity except for in the case of P3 (Figure 3E, dark gray symbols), who was the oldest subject with a macular atrophy (Figure 1B).

### 2.4. Spatial Vision Driven by S Cones and Rods

**Figure 4:**
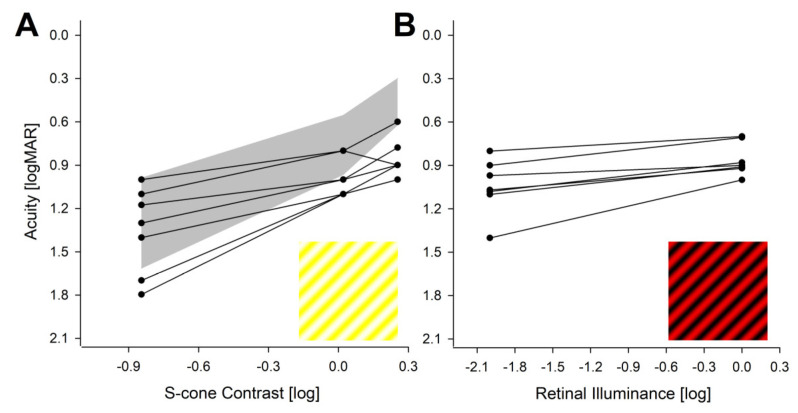
Chromatic visual acuities. (**A**,**B**) Visual acuities in BCM patients recorded with blue on yellow (**A**) and red on black (**B**) gratings. Gray region in panel (**A**) is the normal range. Insets demonstrate the appearance of the gratings to normal trichromats.

To better understand the spatial vision originating from S cones in BCM retinas, we estimated discrimination thresholds of blue gratings superimposed onto a uniform yellow (L/M cone and rod desensitizing) background. The resulting pattern appeared as white gratings on a yellow background to normal trichromatic eyes (Figure 4A, Inset). For the highest available contrast, acuities ranged from 0.6 to 1.0 logMAR for BCM patients which were mostly outside of the normal range (Figure 4A, gray band) from 0.3 to 0.6 logMAR (Figure 4A). With lower contrast gratings, however, there was greater overlap between most BCM patients and normal discrimination thresholds (Figure 4A). Taken together with the expected desensitization of rod function with the yellow background, it is parsimonious to conclude that BCM patients used their S cones to discriminate the blue grating increments at all tested contrast levels. There was no correlation between S cone acuities and age, implying a lack of support to major S cone loss in BCM patients.

To evaluate L/M cone acuity, we used red gratings on a uniform bright blue background. No BCM patient was able to identify these gratings, as expected from the lack of L/M cone function. Any improvement of long-wavelength sensitivity with gene therapy in BCM would be expected to result in the de novo recognition of these gratings.

The isolation of rod-photoreceptor-driven acuity was more challenging. In the normal subjects, this would have required testing with gratings that are dimmer, and thus invisible to dark-adapted cones, which is beyond the scope of the current work. In BCM patients lacking L/M cone vision however, rod-driven acuities could be easily measured with red gratings on a black background in two ambient conditions. The rod acuity in BCM ranged from 0.8 to 1.4 logMAR under dimmer conditions and from 0.7 to 1.0 logMAR under brighter conditions (Figure 4B). There was no correlation between rod acuities and age, implying a lack of support to major rod loss in BCM patients.

### 2.5. Color Discrimination

**Figure 5:**
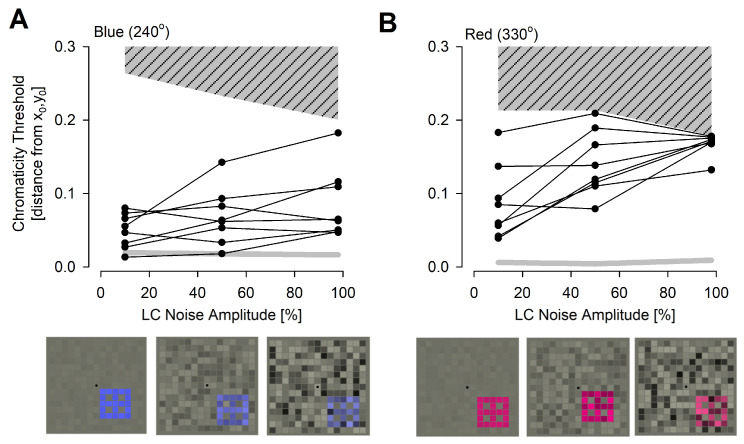
Color vision. (**A**,**B**) Chromaticity thresholds with blue (**A**) and red (**B**) stimuli in BCM patients as a function of LC noise. Hashed region is the color-rendering capability (“phosphor limit”) of the monitor for each color and noise level. The light gray thick line is the normal thresholds. Insets below demonstrate the appearance of the color stimuli embedded in 10%, 50% and 98% LC noise.

BCM patients have been described as showing severe red–green color vision errors along the protan and deutan axes [10,26,27,28]. Color vision along the yellow–blue (tritan) axis of discrimination is thought to be relatively more retained [29] (despite being abnormal) in BCM but recent results suggest an age effect on the severity of the tritan defects [10]. To re-evaluate tritan defects in BCM, chromaticity thresholds for blue hues were measured as a function of luminance contrast (LC) noise amplitude (Figure 5A). For lower LC noise amplitudes (10% and 50%), all BCM patients were able to perform the test and discriminate the stimulus reliably. Thresholds were elevated in all patients except for those of P6, which were near normal. These colored stimuli with lower LC noise amplitude can generate luminance contrast signals and do not necessarily imply perception of color. The highest LC noise amplitude tested (98%), on the other hand, is expected to substantially mask luminance contrast signals and allow blue color signals to drive the discrimination. All BCM patients showed reliable discrimination ability with elevated thresholds at the high LC noise amplitude (Figure 5A). Whether blue discriminations were performed with S cones, light-adapted rods, or via an interaction between the two populations of photoreceptors, was not able to be determined. Chromaticity thresholds to blue at 98% LC noise were highly correlated with age (and pre-retinal absorption) for seven of the eight BCM subjects, consistent with previous results [10]. P3 was an outlier, with better chromaticity thresholds than expected for age.

To confirm severe red color vision defects in BCM and to define potential improvements that may occur upon L-opsin gene therapy, thresholds for red hues were measured as a function of LC noise (Figure 5B). For lower LC noise amplitudes (10% and 50%), all BCM patients were able to perform the test and discriminate the stimulus reliably. Thresholds were elevated in all patients. Considering the lack of L/M cone vision, these results are consistent with the detection of luminance contrast signals mediated by the light-adapted rod system with lower LC noise conditions. At the highest LC noise amplitude tested (98%), no BCM patients (except for one) could reliably perform discrimination, and their thresholds reached the color rendering limit of the monitor (Figure 5B). The exception was P3, who could perform reliable discrimination of the red stimuli with 98% LC noise and this was repeated on three independent sessions performed over two days. P3 reported seeing the stimuli as being darker than the foreground, with no color perception, likely driven by the light-adapted rod system, as this stimulus is not expected to elicit S cone contrast.

### 2.6. Cone Photoreceptor Mosaic

**Figure 6:**
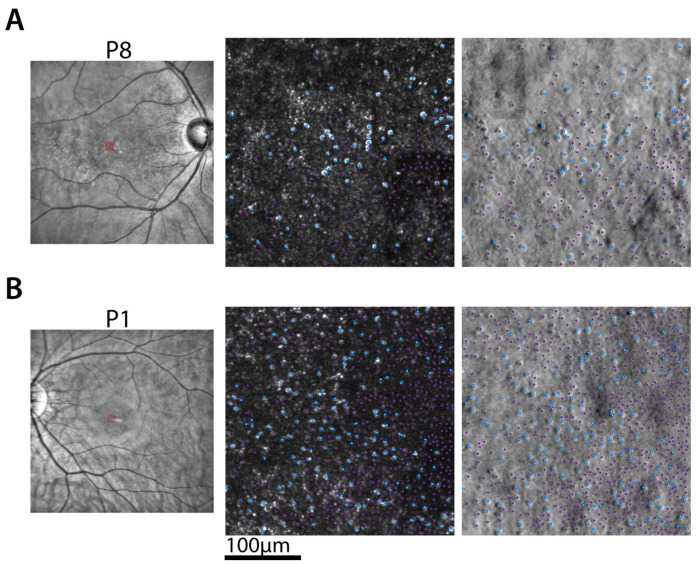
Cone cell densities. (**A**,**B**) NIR SLO and AO confocal and non-confocal images for P8 (**A**) and P1 (**B**). NIR SLO images (left) show the location of the AO images (red squares) and represent the location of peak waveguiding cone density. Confocal (center) and non-confocal split-detection (right) AO images depict the cone mosaic with waveguiding cones marked in blue and non-waveguiding cones marked in purple. Contrast was adjusted on the confocal images for visualization purposes.

To define the distribution and density of cone photoreceptors in BCM retinas, we used AOSLO imaging. Two representative patients (P8 and P1) demonstrated the range of waveguiding cone densities over a 300 µm per side square region with simultaneously acquired confocal and non-confocal split-detection AOSLO imaging (Figure 6). Bright reflections on confocal images, appearing as cones within the photoreceptor mosaic on non-confocal split-detection images, are demarcated with blue dots and considered waveguiding cones. Cones within the non-confocal split-detection images that are non-waveguiding on confocal images are demarcated with purple dots. The region shown is centered at the peak waveguiding cone density over a 55 µm per side square area. P8 demonstrates the low end of the range of densities for waveguiding and total cones (Figure 6A) over the cohort. In contrast, P1 shows more than twice as many waveguiding and non-waveguiding cones compared with P8 (Figure 6B). Across the six BCM patients with successful AOSLO imaging, the distribution and density of cones varied. Waveguiding cone density estimates were 1620, 440, 650, 1650, 370, and 650 cones·mm^−2^ for P1, P2, P5, P6, P7 and P8, respectively. Total cone densities (waveguiding and non-waveguiding cones) over the same areas were 7670, 2890, 7990, 19,740, 7280, and 3060 cones·mm^−2^, respectively. Waveguiding and total cone densities were reduced from normal for all participants. 

Previous work has hypothesized that waveguiding cones are functioning S cones, and non-waveguiding cones are silent L/M cones [3,30,31]. Therefore, we evaluated whether there were correlations between S cone-driven functional measures and waveguiding cone densities. Blue-on-yellow grating acuity measures or mean S cone sensitivities were not correlated with either waveguiding or total cone densities. However, there was a weakly significant linear relationship between the logarithm of chromaticity thresholds to blue CAD stimuli and the logarithm of waveguiding cone densities controlling for age (*p* = 0.03, F-test). This showed a 66% reduction of the chromaticity thresholds score for each ten-fold increase of waveguiding cone density. There were no significant relationships between chromaticity thresholds and non-waveguiding cone densities.

### 2.7. Long-Term Changes

**Figure 7:**
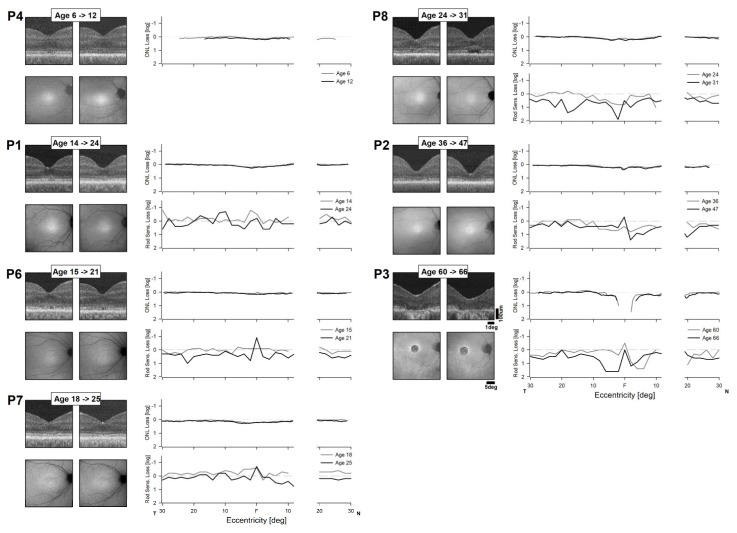
Longitudinal changes in structure and function. Long-term serial data showing NIR-RAFI and OCT images of the central retina (left panels) at two ages in seven of the BCM patients. Additionally shown are the quantification of the ONL thickness (right upper panels) and rod sensitivity loss (right lower panels) at the same ages.

**Figure 8:**
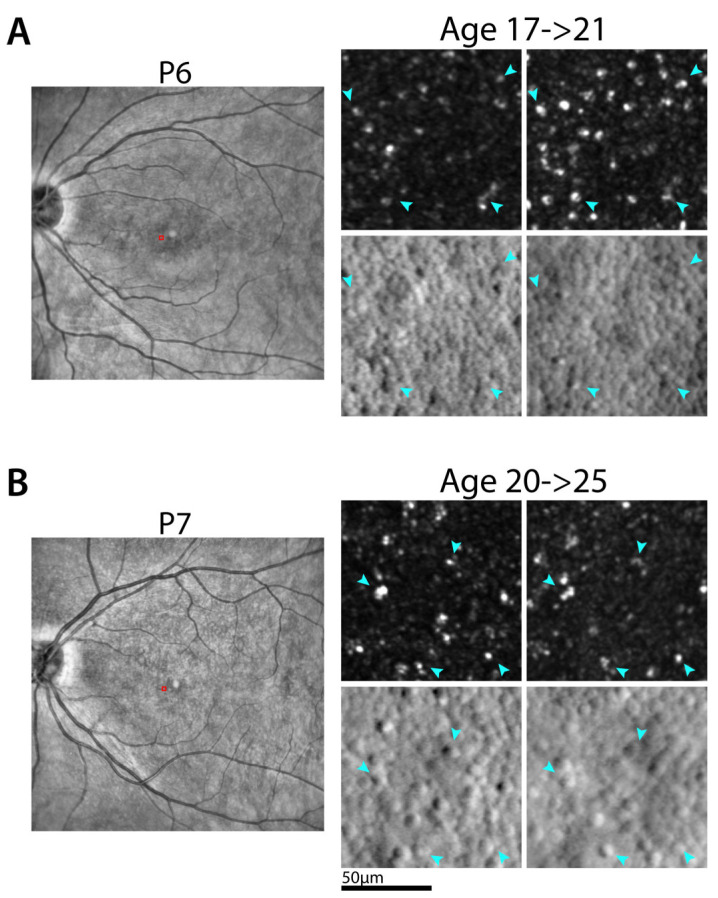
Longitudinal AO images. (**A**,**B**) NIR SLO (left) and AO (right) images for P6 (**A**) and P7 (**B**). NIR images are from the later time point and show the location of the AO images (red squares). AO images are separated by 4.4 and 4.9 years (P6 and P7, respectively). The mosaics are qualitatively stable over this time period. Cones that are waveguiding in the image acquired at the first time point remain waveguiding in the later time point (examples shown by cones labeled with blue arrowheads). Contrast was adjusted on the confocal images for visualization purposes.

Changes in retinal structure and function obtained in longitudinal observations may provide useful information on BCM disease natural history, which tends to progress slowly. In 7 of 8 patients, results were available from 7 years earlier (Figure 7). Qualitatively, on foveal OCT, there were minor changes (P4, P6, P7), loss of IS/OS layer (P1, P2), formation of optical gap (P8), or expansion of atrophy (P3). On NIR-AF there were few, if any, observable changes. Quantitatively, ONL thickness showed no changes, but rod sensitivity losses appear to increase in most patients with age (Figure 7). Longitudinal AO images were available for two patients (P6, P7). AO montages of the photoreceptor mosaic appeared qualitatively stable, though distortions from scanning and eye motion are present within the montages, thereby precluding cell-by-cell alignments over large retinal areas. Cell-by-cell alignments were possible over smaller local regions (Figure 8) and show stability in the mosaic over the time period available. Waveguiding cones in the first time point generally remained waveguiding on follow up. 

## 3. Discussion

BCM patients lack the fine resolution acuity and color vision normally afforded by L/M cone photoreceptor-based day vision but retain good rod photoreceptor-based and S cone photoreceptor-based vision [3,4,5,6,7,8,9,10]. The overarching goal of any BCM therapy is to provide better long-wavelength (orange to red) sensitivity under day vision conditions. Ideally, a therapy would reactivate all silent foveal and extrafoveal photoreceptors and their synapses to connect them to the visual brain. There are also many less-than-ideal outcomes that could still improve vision for BCM patients. For example, a BCM therapy could supplant already active and connected photoreceptors with greater long-wavelength sensitivity. ADVM-062 is a gene augmentation therapy product developed to express the human L-opsin [17]. ADVM-062 is based on the AAV.7m8 capsid intended to be intravitreally administered and uses the MNTC promoter to limit expression to L/M cones. Pre-clinical evaluations supported relative safety of the vector and there was augmentation of long-wavelength function at the level of the retina [17]. Although L-opsin expression was cone-specific and showed greatest transduction of foveal L/M cones, there was also evidence of exogenous L-opsin expression, not only in extrafoveal L/M cones but also in S cones of non-human primate eyes [17]. This unexpected finding set up two potential non-exclusive paths for long-wavelength signals to reach higher vision centers: (1) reactivation of congenitally silent L/M cones, and (2) conversion of a subset of active S cones into novel expanded-wavelength-sensitive photoreceptors. Both paths would likely have distinct advantages and challenges in terms of obtaining meaningful improvements of long-wavelength vision in a future clinical trial using ADVM-062. Current work is aimed at the development of clinical trial outcomes that are based on commercially or publicly available equipment so as to quantify and localize spectrally distinct visual perception at foveal and extrafoveal locations mediated by specific photoreceptors. There has been no distinction made between the two types of mutations, as the number of patients were limited and our previous work [3,4,5,6,7,8,9,10] has shown that the two mutations cannot be distinguished with functional measures. 

### 3.1. Macular Light Sensitivity

At a basic level, the function of all photoreceptors is to signal light by absorbing photons which activate a biochemical cascade. With a gene augmentation approach, improvements to BCM patients’ high-level vision, such as in visual acuity, reading speed, and color vision, can only be achieved by first correcting the primary defect and increasing light sensitivity of the photoreceptors. Perimetric methods are one of the more convenient approaches to map light sensitivity across the retina [32] and they could be acceptable to serve as endpoints in pivotal trials [33,34,35]. The most important part of the retina is the fovea and central macula which provides the largest input to the visual cortex [36]. Within the central retina, BCM patients can have a spectrum of structural defects [3,7,37,38]. However, standard clinical methods for testing light sensitivity in the central retina are often not specific to the photoreceptor type, do not localize function precisely, or both. To understand BCM macular function, we have previously used perimetric methods [3,4,5,6,8,9,10], albeit with specialized equipment that were not easily replicable in future multi-center clinical trials. In the current work, we used two perimeters that are widely available commercial turnkey systems: the free-viewing MonCV1 perimeter [39,40,41,42] and the retina-tracking scotopic MAIA microperimeter [43,44]. The perimeter has the advantage of a wider spectral range of stimuli and chromatic background choices, providing a larger effective dynamic range, minimizing floor and ceiling effects. In patients lacking stable fixation such as BCM, however, the exact retinal localization of the measurements is challenging. The microperimeter uses retinal imaging-based tracking to localize stimuli within the macula but has limited stimulus choices and a limited effective dynamic range that may result in floor effects in more severe stages of the disease.

Results obtained with both perimetric approaches in the current work support and extend the findings of previous work, showing a large deficit of red sensitivity observed under dark-adapted conditions in the central macula of BCM patients [4]. To begin to understand these results, it is important to note the difference between two related concepts which have wrongly been used interchangeably in some of the literature. “Dark-adapted” testing refers to the state of adaptation of the eye and does not necessarily imply which photoreceptors are mediating the perception at a given retinal location. “Scotopic” results, on the other hand, refer to the activity of the rod photoreceptors that provide perception independent of the adaptation state of the eye. In healthy dark-adapted eyes at extramacular locations, most perimetric stimuli are scotopically detected—thus the confusion. However, that is not necessarily the case in the central macula of healthy eyes or at any retinal location in retinal disease. With the use of chromatic differences in so-called scotopic MAIA, we showed that red stimuli within the central 8° diameter region are perceived by L/M cones in normal eyes but by rods in BCM eyes. Near the fovea, the difference in sensitivity between BCM and normal reached 0.5 to 1.5 log or more, providing the magnitude of maximum improvement possible after an intervention such as gene therapy. The use of red stimuli in the central macula has the additional advantage of ensuring that the results are independent of macular pigment density. Macular pigment absorbs in the short-wavelength regime and confounds testing with blue stimuli. Macular pigment naturally varies between individuals with healthy retinas, and there are further contributions among retinal degenerations affecting the fovea and its immediate surrounds [45,46].

### 3.2. Extramacular Light Sensitivity

As intravitreal gene therapy administration can potentially transfect cells across the retina, it is paramount to sample light sensitivity of the extramacular retina for considerations of both safety and efficacy. In terms of safety, retained rod and S cone function in BCM could be reduced; in terms of efficacy, the lacking function that is mediated by long-wavelength-sensitive opsin could be recovered. Automated perimetry is an important outcome measure that can sample light sensitivity; however, as exemplified in the current cohort, patients younger than 12 years old may not always perform reliably. Perimetry is a valid surrogate endpoint for clinical trials that is acceptable to regulatory agencies [33,34,47]. Specifically, an average change of 7 dB or more at five or more pre-specified locations is thought to be clinically significant and potentially approvable. In healthy eyes, standard automated perimetry performed under light-adapted conditions is mediated by L/M cone photoreceptor function. However, in BCM, a combination of S cones and rods mediates the responses [4]. Sensitivity to standard white stimuli on standard white-light-adapting conditions was abnormally reduced at all extramacular locations in all BCM patients. Thus, any potential positive and negative changes can be evaluated with standard automated perimetry but there can be many confounders, and interpretation of the results will not be possible when there may be a mix of both efficacy and subclinical toxicity.

Rod and S cone specific function was measured using short-wavelength (500 and 440 nm, respectively) stimuli. Results are consistent with previous work [3,4] showing near normal sensitivity. However, spectral sensitivity functions showed a relatively large confounder driven by age-related increase of the pre-retinal absorption of short-wavelength lights driven mostly by lenticular yellowing [24,48]. Individual contributions to the pre-retinal absorption, ranging from 0.3 to 1.3 log units, could be defined for the first time using a turnkey commercial instrument which has not been possible in the past. Thus, any potential deleterious effects to the retained rod and S cone function in BCM due to an intravitreal injection can be evaluated quantitatively with perimetry. Importantly, spectral results confirm long-wavelength stimuli as being perceived by the rod system in extra-macular BCM retina, requiring a different approach to detect any increase in exogenous L-opsin mediated sensitivity. This was not attempted in the current work, but future studies could evaluate photoreceptor-specific conditions, such as presenting red stimuli on a blue adapting background.

### 3.3. Color Vision

Severe color vision deficit in BCM is intuitively understandable due to complete lack of L/M cone function. What is less clear is whether BCM patients retain any color signals. Normal color vision is more than just the detection of different wavelengths and requires a comparison between multiple cone inputs. It is, however, possible that the sensory cortex adapts to congenital input from only rods and S cones to provide a rudimentary color vision signal. Using color cap arrangement tests based on Farnsworth–Munsell 100 Hue, we and others have found greater errors along the protan and deutan axes and relative retention along the tritan axis in BCM [10,26,27,28]. BCM patients were also able to discriminate shorter wavelengths [29]. To better isolate different color signals and reduce detection by luminance contrast signals, we also previously used color assessment and diagnosis (CAD), which showed an unexpected variation in chromaticity thresholds along the yellow–blue axis, with an apparent worsening with age [10]. Current work with CAD but with an extended protocol also showed blue chromaticity thresholds to be highly correlated with age for the majority of the BCM patients. Since short-wavelength sensitivity was also found to be age-related through the increase in pre-retinal absorption, it is parsimonious to assume that the age relation of the blue color vision defect was similarly affected. Most important for a potential efficacy signal driven by an exogenous L-opsin was red color vision, which was found to be severely defective in all BCM patients. When a future intervention is successful in achieving an increase in long-wavelength sensitivity as a result of the expression of exogenous L-opsin, the CAD test could be used to evaluate whether such an improvement results in a novel color discrimination ability along the red–green axis.

### 3.4. Spatial Vision

The Early Treatment of Diabetic Retinopathy Study (ETDRS) chart and procedures of measuring BCVA have together formed the gold standard for determining spatial resolving capacity in human eyes [33,49]. Despite this pinnacle of success, ETDRS-BCVA has several shortcomings in terms of understanding the consequences of disease progression and treatment. First, fixed charts cause obvious practice/learning effects when visual acuity measurements are repeated as typically specified in first-in-human protocols. To avoid memorization, different ETDRS charts may be used, though choices are limited. Second, it is very difficult to uniformly apply the ETDRS method in patients with acuities that fluctuate near 20/200. Different test distances (1, 2 and 4 m) may be used to estimate distance-corrected acuities. However, changing distances (and compensating refraction), particularly in younger subjects, makes it very difficult to reliably and reproducibly record small changes in BCVA. Finally, the identity of the photoreceptors mediating BCVA with the standard achromatic method is unknown. In healthy eyes, L/M cones mediate acuity in daylight conditions. In BCM eyes on the other hand, S cones and light-adapted rod cells combine to mediate the BCVA.

Electronic charts eliminate learning effects by randomizing the presentations, allowing chromatic combinations with the potential for a greater specificity of photoreceptor sources and covering a large acuity range at a single distance with high-resolution and large monitors [50,51]. We chose to use a public domain software [52] and full screen diagonal chromatic gratings instead of optotypes to allow patients with vision loss to identify the direction of gratings easily instead of needing to first find the optotype and then identify it. As expected from our previous work using a custom-modified microperimeter [6,7], no BCM patients perceived any red gratings presented on a bright blue background, which is expected to desensitize both rod and S cone photoreceptors. Any potential perceptual improvements driven by an exogenous L-opsin would be expected to result in the visibility of the red-on-blue gratings and provide a photoreceptor-specific estimate of the spatial vision possible. 

To evaluate the potential subclinical toxicity of a future intervention to retained rods and S cones of BCM patients, we used red gratings under low-luminance conditions and blue gratings on bright yellow backgrounds, respectively. Spatial vision in both cases tended to be lower than ETDRS-BCVA, likely as a consequence of the specialized adapting conditions. It is important to note that grating acuities were performed with natural pupils and thus the rod desensitization of the bright yellow background may not have been complete. Future work will have to evaluate these conditions in complete achromats to detect contributions to spatial vision driven by light-adapted rods. Nevertheless, blue gratings were likely driven by the activity of retained S cones in BCM patients. Similarly, red gratings under low luminance were likely driven by rods in BCM patients. However, comparative estimates of rod-driven spatial vision in healthy eyes will require future work with lower levels of luminance.

### 3.5. Cone Photoreceptor Identity and Density

Use of adaptive optics to compensate for the eye’s aberrations has revolutionized our ability to image the photoreceptor mosaic and quantify the density of photoreceptors [53,54]. Early results in deuteranopes with M-opsin mutations showing gaps in the cone mosaic were interpreted as missing cones [55]. In BCM patients with L/M-opsin mutations, the earliest AOSLO imaging showed waveguiding bright cones and dysreflective dark cones, which are hypothesized to potentially represent functional S cones and dysfunctional but surviving L/M cones, respectively [3,38]. The development of split-detection AOSLO has allowed the direct demonstration of non-waveguiding cones with retained inner segments (and thus cone cells) lacking normal outer segments in achromatopsia and BCM [30,31,56]. The current work also supports previous work. The direct identification of all waveguiding cones as being only S cones remains elusive to date. The current work has provided evidence of a correlation between blue chromaticity thresholds and waveguiding cone densities and longitudinal imaging has revealed the stability of waveguiding cones, thus indirectly supporting the hypothesis.

### 3.6. Likelihood of Vision Improvement in BCM

BCM falls into the category of IRDs characterized by a dissociation between function and structure, with evidence of retained but dysfunctional photoreceptors that have significant potential for improvement [57]. BCM has not been treated to date, but interventions performed in other members of this category could be helpful to estimate the spectrum of outcomes resulting from gene therapy. Very large rod-mediated improvements recorded in adults following subretinal gene therapy in *RPE65*-LCA and *GUCY2D*-LCA suggest that dormant rod photoreceptors can be awakened after apparently retaining all of the required molecular machinery for decades [58,59]. Foveal L/M cone sensitivity has also been shown to improve in *CEP290*-LCA following intravitreally administered antisense oligonucleotide treatment or subretinally injected gene editing therapy [60,61,62]. Most closely related to BCM are achromatopsia (ACHM) trials. In both *CNGA3*- and *CNGB3*-ACHM, subretinal gene therapy has shown rare and minor improvements that can possibly be linked to improved cone function [63,64,65,66,67]. A clinical trial with intravitreal CNTF in *CNGB3*-ACHM did not show improved cone function [68]. Unlike ACHM, however, BCM does not lack all cone input to the visual cortex and thus may have a better chance of perceiving the novel long-wavelength signals introduced with gene augmentation.

### 3.7. Proposed Outcomes for a Clinical Trial 

Successful proof-of-concept studies of intravitreal ADVM-062 in animals need to be followed by the submission of investigational and new-drug-enabling study results to regulatory authorities before a first-in-human early-phase clinical trial can be initiated for BCM. A key primary outcome for such a future trial will be the clinical exam for evaluating safety. Other safety-related outcomes may include OCT and en face imaging such as SW-RAFI and NIR-RAFI [69] to evaluate sub-clinical changes to the photoreceptors and the retinal pigment epithelium. Early stages of disease in BCM retinas tend to show minor disturbances on OCT at the layer of photoreceptor outer segments [3,7] which may possibly change further in response to an intervention. The current work has shown that different methods, such as temporal (during acquisition) versus spatial (after acquisition) averaging, may show different sensitivity to minor and heterogenous changes. The scanning speeds for OCTs have increased dramatically over the years and higher speeds are likely to reduce artefactual smoothing effects of temporal averaging in eyes with unsteady fixation.

In terms of functional measures of safety and efficacy, standard BCVA and standard automated perimetry are both abnormally reduced in all BCM patients and can potentially show both decrements (toxicity) and increments (efficacy) from baseline after an intervention. However, these standard tests are not very specific and there are many potential considerations where they may not be sensitive to change. Therefore, we considered outcome measures that were more specific to different photoreceptor populations in the current work. Previous results in congenitally blind eyes of *CEP290*-LCA, which demonstrated light sensitivity improvement of cones without acuity improvements following treatment [70], imply that most important outcomes of early phase trials in BCM will be based on perimetry. Our results support the scotopic MAIA microperimetry, with a red stimulus sampling of the foveal and parafoveal regions as the key secondary endpoint to evaluate the efficacy of the exogenous L-opsin expression at or near the dormant foveal L/M cones that are the main targets of the intravitreal ADVM-062 vector. Real-time tracking of the retina with microperimetry would allow careful localization and interpretation of the visual function results, and substantial reduction of red thresholds in all BCM patients would allow for a large range of measurable improvement. Once there is evidence of the improvement of light sensitivity, another outcome measure based on red gratings on a bright blue background would allow evaluation of spatial vision specific to L-opsin.

The fovea/macula region of BCM demonstrates slow progressive degeneration [7] and L-opsin gene therapy directed to the fovea would need to be timed to a stage of disease at which there is still evidence of retained-but-dysfunctional L/M cones. However, any progression at the extrafoveal retina of BCM patients is less obvious and thus potentially provides a much wider therapeutic window if L-opsin expression can be achieved outside of the fovea.

## 4. Materials and Methods

### 4.1. Patients 

Eight patients were enrolled prospectively; three had large structural variants at the OPN1LW/OPN1MW gene cluster, and five patients carried the p.C203R missense variant in single or multiple OPN1LW/OPN1MW genes (Table 1). Patients underwent a complete ophthalmic examination, including BCVA using standard high contrast (black letters on white background) back-illuminated charts with standard ETDRS methodology [49]. In 7 of 8 patients, long term serial data were available (median follow-up 6.5 years, range 6–11 years). All functional measures obtained in the current study were designed to be equally applicable to all severity stages of BCM; however, perceptual measures, such as perimetry, can sometimes be unreliable in patients younger than 12 years old.

### 4.2. En Face and Cross-Sectional Imaging 

En face imaging was obtained with a confocal scanning laser ophthalmoscope (Spectralis HRA, Heidelberg Engineering, Heidelberg, Germany) using NIR reflectance and NIR-RAFI modalities [3,7,8]. Cross-sectional imaging was obtained with two spectral-domain OCT devices. A Spectralis HRA + OCT device (Heidelberg Engineering, Heidelberg, Germany) was used to obtain 30° long line scans with a high-speed mode (768 a-scans) 30° lens and automatic real time (ART) setting set to 20 providing temporal averaging. Axial resolution was estimated to be ~8 µm and was sampled at 3.87 µm. Additional OCT imaging was performed with an RTvue-100 device (Optovue Inc., Fremont, CA, USA) to obtain 30° long single high-density (HD-Line) scans, which were then spatially averaged [3,7,8]. Axial resolution was estimated to be ~5 µm and was sampled at 3.01 µm. OCT data were exported from each device and analyzed with custom computer programs (MatLab 2023a; MathWorks Inc., Natick, MA, USA).

### 4.3. Perimetry, Spectral Sensitivity, and Microperimetry

The primary function of photoreceptors is to detect changes in light levels, and free-viewing (standard) perimetry and retina-tracking perimetry (microperimetry) provide complementary approaches to sampling light sensitivity across the visual field. In general, free-viewing perimetry provides stimulus choices with a substantially greater range of luminance and colors that can be presented anywhere across the visual field, whereas retina-tracking perimetry provides better retinal localization in subjects with unstable fixation. Free-viewing perimetry was performed with a computerized perimeter (MonCVOne, Metrovision, Perenchies, France) which can perform dark-adapted chromatic, standard light-adapted achromatic, and S cone testing without modification [39,71]. Methods were similar to those previously described [4,32,39,71,72] and measurements were performed at 2° intervals across the visual field, extending to 30° eccentricity from the fixation along horizontal and vertical meridians with Goldmann V size (1.7° diameter) and 200 ms duration stimuli in eyes with dilated pupils. Dark-adapted sensitivities were measured with monochromatic blue-green (500 nm) and red (650 nm) stimuli. Light-adapted sensitivities were measured with white stimuli on a white (10 cd.m^−2^) background and with monochromatic violet (440 nm) stimuli on a yellow (100 cd.m^−2^) background. In a subset of five patients (P1, P2, P3, P5, and P8), spectral sensitivity functions were obtained with four monochromatic stimuli (440, 500, 600, and 650 nm) at three neighboring retinal locations in the superior and inferior visual fields (12° eccentric) with methods previously described [3,73,74,75]. All available chromatic sensitivities were ensemble fit, with rod (dark-adapted) and S cone (yellow light-adapted) photoreceptor sensitivities adjusted for individualized pre-retinal absorption [24,48,76,77].

Retina-tracking perimetry (S-MAIA, Scotopic Macular Integrity Assessment, CenterVue, Padova, Italy) was performed after 45 min of dark-adaptation. The test pattern was a custom cross centered on the anatomical fovea and extending along each principal meridian to 4° eccentric at 0.5° steps with additional loci placed at 6° eccentric. Testing was undertaken with a 4–2 dB staircase strategy with cyan (505 nm) and red (627 nm) stimuli of Goldmann III (0.43° diameter) size and 200 ms duration. The background was dim red. Before the start of the testing, OCT information was used to depict the individualized location of the anatomical foveal depression and major blood vessels on a transparency printed at scale. At the start of the perimetric testing, this transparency was used to manually adjust the center of the test grid to the center of the anatomic fovea. This approach was necessary, as some patients did not fixate at the fovea and the exact foveal location was often not reliably recognizable with the imaging available during microperimetric testing. All microperimetric tests were repeated with the follow-up mode and the results were averaged.

### 4.4. Spatial Vision with Grating Acuities

To evaluate spatial vision, chromatic gratings were presented with the Freiburg Visual Acuity and Contrast Test 10 (FrACT10, ver. 1.0.6) [52] on a fully calibrated, 10 bit 24” monitor (ColorEdge CS2420, EIZO, Hakusan, Ishikawa, Japan) placed at 2 m distance (see Supplementary Figure S2 for software setup used). The distance, monitor size, and pixel pitch provided a ~100-fold range of spatial frequencies from 0.12 cpd (to allow at least two bars to be visible) to 15.04 cpd (to avoid aliasing) corresponding to Snellen equivalents of 20/5120 to 20/40 or 2.40 to 0.30 logMAR (30 cpd = 0 logMAR). The approach was similar to our previous work with a custom-built system with Maxwellian optics [6,7,59], with the exception of the use of a free viewing setup with no dilation, commercial equipment, and public domain software, which allowed for easier replication. Diagonal gratings filled a rectangular area subtending 15° × 8.6°, and testing was performed with a two alternative forced choice paradigm in a dark room. Acuity was estimated using the method of limits starting from easily visible spatial frequencies and incrementing in 0.1 logMAR steps similar to ETDRS. At each step, 10 trials were performed; the stop criterion was the subject obtaining less than 90% correct responses. Starting spatial frequency value was chosen based on ETDRS acuity, and the finest grating detected with ≥90% correct responses was taken as the grating acuity. The refractive correction used was the same as ETDRS testing.

To differentiate between types of photoreceptors dominating perception of gratings, different chromaticities and luminances were used. To estimate S cone-mediated acuity (in BCM patients and normal subjects), increments of blue gratings (max 10 phot-cd.m^−2^ or 181 scot-cd.m^−2^) were presented on an invariant bright-yellow-adapting background (117 phot-cd.m^−2^ or 208 scot-cd.m^−2^). In addition to the maximal available grating increment, two lower increments were also used corresponding to a range of S cone Michaelson contrasts from 180% to 14%. To estimate L/M cone-mediated acuity (in BCM patients and normal subjects), a maximal increment of red gratings (34 phot-cd.m^−2^ or 4.5 scot-cd.m^−2^) was presented on a bright-blue-adapting background (10 phot-cd.m^−2^ or 181 scot-cd.m^−2^). To estimate rod-mediated acuity (in BCM patients), red gratings were presented on a black background under direct viewing, and additionally through a 2-log unit neutral density filter after a period of dark adaptation for at least 10 min. Rod-mediated acuity in normal subjects was not measurable with the current protocol.

### 4.5. Color Vision

The CAD test (ver.2.6.1, City Occupational Ltd., Cumbria, UK—now COL-AEGLIA Institute for Occupational Vision, Lelystad Airport, Netherlands) was used to quantify color vision impairment. The visual stimuli were generated on a fully calibrated, 10-bit 24” monitor (ColorEdge CS2420, EIZO, Hakusan, Ishikawa, Japan) [78]. The standard CAD test protocol [79] was adapted for use with low vision patients [10] by tripling the size of the stimulus (physical viewing distance of 1 m, but retaining a software setting of 2.8 m, see Supplementary Figure S3 for software setup used). The test employed three nesting regions: a larger background, an intermediate foreground, and a small stimulus. The background had a 19° angular subtense and 24 cd.m^−2^ luminance. The CIE chromaticity of the background (0.305, 0.323) was close to the chromaticity of the CIE illuminant D65. Centered within the uniform background was the foreground subtending 8° and divided into a 15 × 15 square array of checks flickering randomly in luminance at 12 Hz. The range of luminance the program selected for every check in the array was determined by the LC noise amplitude specified between 0% and 100%. For example, for an LC noise amplitude of 50% and a background luminance of Lb, the luminance of any check was selected randomly, with an equal probability between 1.5×Lb and 0.5×Lb. An important outcome of this technique was that the mean luminance of each check during the presentation and the mean luminance over the entire foreground remained unchanged and equal to the background luminance.

Within the foreground was a colored stimulus subtending 2.7° divided into a 5 × 5 square array of checks flickering randomly. Each test run consisted of the stimulus appearing near one of the four corners of the foreground and moving diagonally towards the opposite corner at 8°/s. The testing method was the four alternative forced choice (4AFC) method, with an auditory prompt. Each session consisted of many tests, in which the color signal strength is controlled by a staircase procedure according to the patient’s response. If the patient correctly identified the direction of the moving stimulus twice during sequential presentation, the algorithm reduced the chromatic saturation of the stimulus, bringing its chromaticity closer to that of the background to make it more difficult to see. When the patient failed to produce a correct response, the chromatic saturation was increased to make it easier to see. Each session consisted of 11 reversals and the threshold for the session was calculated by averaging the last 6 reversals. There were six sessions of testing consisting of three LC noise amplitudes (10%, 50% or 98%, starting with the lowest and ending with the highest) and two chromaticities. One of the stimuli was at the chromaticity angle of 240°, with a blue hue chosen in order to generate a large S cone contrast. The other stimulus was at the chromaticity angle of 330°, with a red hue chosen to generate a large L-cone contrast but to remain isoluminant with the gray fore- and background in terms of S cones. Both chromaticies had the potential to produce residual scotopic/rod contrasts. Display limits available along each chromatic angle were measured experimentally by a subject with normal vision, intentionally providing the wrong response for each presentation.

With low LC contrast amplitudes, both normal trichromats and BCM subjects were able to obtain reliable thresholds that were well within the display limits by performing the test using either luminance or chromatic contrast. Previous studies have shown that high amplitude LC noise tends to mask the use of residual luminance contrast signals present in colored stimuli that are designed to be ‘isoluminant’ for the CIE standard observer [80]. Thus, as LC noise amplitudes approach 100%, subjects are expected to need greater contributions from the chromatic signal to correctly judge its direction of motion; however, the completeness of isolation from scotopic luminance signals in severe loss of cone function remains to be evaluated. In normal trichromats, when the color signal strength is at or above the subject’s color threshold, any further increase in LC noise has little effect on the subject’s color threshold.

### 4.6. Adaptive Optics (AO) Imaging

To image the photoreceptor mosaic and estimate the density of cone photoreceptors, we used a custom-built, multimodal adaptive optics scanning laser ophthalmoscope (AOSLO) system, previously described in [56,81,82]. Three photomultiplier tubes (Hamamatsu Corporation) were configured to simultaneously record confocal and non-confocal split-detection near-infrared reflectance image sequences at 18 Hz. AOSLO image sequences were acquired over the central 3° surrounding the anatomical fovea, and along superior and temporal meridia until reaching approximately 15° eccentricity. AOSLO image sequences from each retinal location were desinusoided, registered [83], and semi-automatically montaged [84]. To quantify, a region was first selected at or near the anatomical fovea that appeared to contain the highest density of waveguiding cones. One grader (R.L.W.) manually identified the waveguiding cones within this region. A sliding square window 55 µm per side was then used to calculate the waveguiding cone density at each pixel and the location of peak waveguiding cone density was determined. Non-waveguiding cones within the 55 µm square region of interest centered on the location of peak waveguiding density were manually identified. All waveguiding and non-waveguiding cones were then considered when calculating total cone density. Waveguiding and non-waveguiding cones were also identified over a 300 µm per side square centered on the peak waveguiding cone location and waveguiding and total cone density was calculated over this region. AOSLO imaging was successful in six of eight patients (all but P3 and P4).

## Figures and Tables

**Table 1:** Characteristics of BCM patients.

ID	Age [years]	Genotype	BCVA ^1^ [logMAR]	Refraction	Axial Length [mm]
P1	23	Deletion LCR	0.94	Plano	26.0
P2	46	Deletion LCR	0.66	−6.25 −2.00 × 155	26.5
P3	66	Deletion LCR	0.90	−5.00 DS	na
P4	11	C203R	0.84	−11.50 +3.50 × 094	27.7
P5	19	C203R	0.82	Plano −1.00 × 050	25.0
P6	21	C203R	0.62	−3.75 −3.25 × 165	25.8
P7	25	C203R	0.60	−3.25 −0.75 × 000	26.2
P8	30	C203R	0.76	−5.25 −1.50 × 040	25.7

^1^ Best corrected visual acuity.

## Data Availability

The data presented in this study are available on request from the corresponding author due to patient confidentiality.

## Supplementary Materials

The following supporting information can be downloaded at https://www.mdpi.com/article/10.3390/ijms251910639/s1, Figure S1: Comparison of temporal vs. spatial averaging on outer retinal subcellular defects; Figure S2: Setup parameters used in the public domain web-based FrACT10 application to measure chromatic acuities with diagonal gratings; Figure S3: Setup parameters used in the commercial CAD application to measure color vision.

## References

[1] Nathans J., Davenport C.M., Maumenee I.H., Lewis R.A., Hejtmancik J.F., Litt M., Lovrien E., Weleber R., Bachynski B., Zwas F. (1989). Molecular genetics of human blue cone monochromacy. Science.

[2] Wissinger B., Baumann B., Buena-Atienza E., Ravesh Z., Cideciyan A.V., Stingl K., Audo I., Meunier I., Bocquet B., Traboulsi E.I. (2022). The landscape of submicroscopic structural variants at the OPN1LW/OPN1MW gene cluster on Xq28 underlying blue cone monochromacy. Proc. Natl. Acad. Sci. USA.

[3] Cideciyan A.V., Hufnagel R.B., Carroll J., Sumaroka A., Luo X., Schwartz S.B., Dubra A., Land M., Michaelides M., Gardner J.C. (2013). Human cone visual pigment deletions spare sufficient photoreceptors to warrant gene therapy. Hum. Gene Ther..

[4] Luo X., Cideciyan A.V., Iannaccone A., Roman A.J., Ditta L.C., Jennings B.J., Yatsenko S.A., Sheplock R., Sumaroka A., Swider M. (2015). Blue cone monochromacy: Visual function and efficacy outcome measures for clinical trials. PLoS ONE.

[5] Roman A.J., Cideciyan A.V., Matsui R., Sheplock R., Schwartz S.B., Jacobson S.G. (2015). Outcome measure for the treatment of cone photoreceptor diseases: Orientation to a scene with cone-only contrast. BMC Ophthalmol..

[6] Cideciyan A.V., Roman A.J., Jacobson S.G., Yan B., Pascolini M., Charng J., Pajaro S., Nirenberg S. (2016). Developing an outcome measure with high luminance for optogenetics treatment of severe retinal degenerations and for gene therapy of cone diseases. Investig. Ophthalmol. Vis. Sci..

[7] Sumaroka A., Garafalo A.V., Cideciyan A.V., Charng J., Roman A.J., Choi W., Saxena S., Aksianiuk V., Kohl S., Wissinger B. (2018). Blue cone monochromacy caused by the C203R missense mutation or large deletion mutations. Investig. Ophthalmol. Vis. Sci..

[8] Sumaroka A., Cideciyan A.V., Sheplock R., Wu V., Kohl S., Wissinger B., Jacobson S.G. (2020). Foveal therapy in blue cone monochromacy: Predictions of visual potential from artificial intelligence. Front. Neurosci..

[9] Semenov E.P., Sheplock R., Roman A.J., McGuigan D.B., Swider M., Cideciyan A.V., Jacobson S.G. (2020). Reading performance in blue cone monochromacy: Defining an outcome measure for a clinical trial. Transl. Vis. Sci. Technol..

[10] Mascio A.A., Roman A.J., Cideciyan A.V., Sheplock R., Wu V., Garafalo A.V., Sumaroka A., Pirkle S., Kohl S., Wissinger B. (2023). Color vision in blue cone monochromacy: Outcome measures for a clinical trial. Transl. Vis. Sci. Technol..

[11] Smallwood P.M., Olveczky B.P., Williams G.L., Jacobs G.H., Reese B.E., Meister M., Nathans J. (2003). Genetically engineered mice with an additional class of cone photoreceptors: Implications for the evolution of color vision. Proc. Natl. Acad. Sci. USA.

[12] Mancuso K., Hauswirth W.W., Li Q., Connor T.B., Kuchenbecker J.A., Mauck M.C., Neitz J., Neitz M. (2009). Gene therapy for red-green colour blindness in adult primates. Nature.

[13] Zhang Y., Deng W.T., Du W., Zhu P., Li J., Xu F., Sun J., Gerstner C.D., Baehr W., Boye S.L. (2017). Gene-based therapy in a mouse model of blue cone monochromacy. Sci. Rep..

[14] Ma X., Sechrest E.R., Fajardo D., Zhu P., Dyka F., Wang Y., Lobanova E., Boye S.E., Baehr W., Deng W.T. (2022). Gene Therapy in Opn1mw(-/-)/Opn1sw(-/-) mice and implications for blue cone monochromacy patients with deletion mutations. Hum. Gene Ther..

[15] Deng W.T., Li J., Zhu P., Freedman B., Smith W.C., Baehr W., Hauswirth W.W. (2019). Rescue of M-cone function in aged Opn1mw-/- mice, a model for late-stage blue cone monochromacy. Investig. Ophthalmol. Vis. Sci..

[16] Deng W.T., Li J., Zhu P., Chiodo V.A., Smith W.C., Freedman B., Baehr W., Pang J., Hauswirth W.W. (2018). Human L- and M-opsins restore M-cone function in a mouse model for human blue cone monochromacy. Mol. Vis..

[17] Hanna K., Nieves J., Dowd C., Bender K.O., Sharma P., Singh B., Renz M., Ver Hoeve J.N., Cepeda D., Gelfman C.M. (2023). Preclinical evaluation of ADVM-062, a novel intravitreal gene therapy vector for the treatment of blue cone monochromacy. Mol. Ther..

[18] Dalkara D., Byrne L.C., Klimczak R.R., Visel M., Yin L., Merigan W.H., Flannery J.G., Schaffer D.V. (2013). In vivo-directed evolution of a new adeno-associated virus for therapeutic outer retinal gene delivery from the vitreous. Sci. Transl. Med..

[19] Khabou H., Garita-Hernandez M., Chaffiol A., Reichman S., Jaillard C., Brazhnikova E., Bertin S., Forster V., Desrosiers M., Winckler C. (2018). Noninvasive gene delivery to foveal cones for vision restoration. JCI Insight..

[20] Grishanin R., Vuillemenot B., Sharma P., Keravala A., Greengard J., Gelfman C., Blumenkrantz M., Lawrence M., Hu W., Kiss S. (2019). Preclinical evaluation of ADVM-022, a novel gene therapy approach to treating wet age-related macular degeneration. Mol. Ther..

[21] Sahel J.A., Boulanger-Scemama E., Pagot C., Arleo A., Galluppi F., Martel J.N., Esposti S.D., Delaux A., de Saint Aubert J.B., de Montleau C. (2021). Partial recovery of visual function in a blind patient after optogenetic therapy. Nat. Med..

[22] Ross M., Obolensky A., Averbukh E., Desrosiers M., Ezra-Elia R., Honig H., Yamin E., Rosov A., Dvir H., Gootwine E. (2022). Outer retinal transduction by AAV2-7m8 following intravitreal injection in a sheep model of CNGA3 achromatopsia. Gene Ther..

[23] Ail D., Ren D., Brazhnikova E., Nouvel-Jaillard C., Bertin S., Mirashrafi S.B., Fisson S., Dalkara D. (2022). Systemic and local immune responses to intraocular AAV vector administration in non-human primates. Mol. Ther. Methods Clin. Dev..

[24] Charng J., Tan R., Luu C.D., Sadigh S., Stambolian D., Guymer R.H., Jacobson S.G., Cideciyan A.V. (2017). Imaging lenticular autofluorescence in older subjects. Investig. Ophthalmol. Vis. Sci..

[25] Curcio C.A., Sloan K.R., Kalina R.E., Hendrickson A.E. (1990). Human photoreceptor topography. J. Comp. Neurl..

[26] Weiss A.H., Biersdorf W.R. (1989). Blue cone monochromatism. J. Pediatr. Ophthalmol. Strabismus..

[27] Ayyagari R., Kakuk L.E., Bingham E.L., Szczesny J.J., Kemp J., Toda Y., Felius J., Sieving P.A. (2000). Spectrum of color gene deletions and phenotype in patients with blue cone monochromacy. Hum. Genet..

[28] Michaelides M., Johnson S., Simunovic M.P., Bradshaw K., Holder G., Mollon J.D., Moore A.T., Hunt D.M. (2005). Blue cone monochromatism: A phenotype and genotype assessment with evidence of progressive loss of cone function in older individuals. Eye.

[29] Reitner A., Sharpe L.T., Zrenner E. (1991). Is colour vision possible with only rods and blue-sensitive cones?. Nature.

[30] Scoles D., Flatter J.A., Cooper R.F., Langlo C.S., Robison S., Neitz M., Weinberg D.V., Pennesi M.E., Han D.P., Dubra A. (2016). Assessing photoreceptor structure associated with ellipsoid zone disruptions visualized with optical coherence tomography. Retina.

[31] Patterson E.J., Kalitzeos A., Kane T.M., Singh N., Kreis J., Pennesi M.E., Hardcastle A.J., Neitz J., Neitz M., Michaelides M. (2022). Foveal cone structure in patients with blue cone monochromacy. Investig. Ophthalmol. Vis. Sci..

[32] Cideciyan A.V., Krishnan A.K., Roman A.J., Sumaroka A., Swider M., Jacobson S.G. (2021). Measures of function and structure to determine phenotypic features, natural history, and treatment outcomes in inherited retinal diseases. Annu. Rev. Vis. Sci..

[33] Csaky K., Ferris F., Chew E.Y., Nair P., Cheetham J.K., Duncan J.L. (2017). Report from the NEI/FDA endpoints workshop on age-related macular degeneration and inherited retinal diseases. Investig. Ophthalmol. Vis. Sci..

[34] Thompson D.A., Iannaccone A., Ali R.R., Arshavsky V.Y., Audo I., Bainbridge J.W.B., Besirli C.G., Birch D.G., Branham K.E., Cideciyan A.V. (2020). Advancing clinical trials for inherited retinal diseases: Recommendations from the second Monaciano symposium. Transl. Vis. Sci. Technol..

[35] Schmetterer L., Scholl H., Garhofer G., Janeschitz-Kriegl L., Corvi F., Sadda S.R., Medeiros F.A. (2023). Endpoints for clinical trials in ophthalmology. Prog. Retin. Eye Res.

[36] Wandell B.A., Winawer J. (2011). Imaging retinotopic maps in the human brain. Vision Res..

[37] Ayyagari R., Kakuk L.E., Coats C.L., Bingham E.L., Toda Y., Felius J., Sieving P.A. (1999). Bilateral macular atrophy in blue cone monochromacy (BCM) with loss of the locus control region (LCR) and part of the red pigment gene. Mol. Vis..

[38] Carroll J., Dubra A., Gardner J.C., Mizrahi-Meissonnier L., Cooper R.F., Dubis A.M., Nordgren R., Genead M., Connor T.B., Stepien K.E. (2012). The effect of cone opsin mutations on retinal structure and the integrity of the photoreceptor mosaic. Investig. Ophthalmol. Vis. Sci..

[39] Roman A.J., Powers C.A., Semenov E.P., Sheplock R., Aksianiuk V., Russell R.C., Sumaroka A., Garafalo A.V., Cideciyan A.V., Jacobson S.G. (2019). Short-wavelength sensitive cone (S-cone) testing as an outcome measure for NR2E3 clinical treatment trials. Int. J. Mol. Sci..

[40] Simunovic M.P., Hess K., Avery N., Mammo Z. (2021). Threshold versus intensity functions in two-colour automated perimetry. Ophthalmic. Physiol. Opt..

[41] Oertli J.M., Pfau K., Scholl H.P.N., Jeffrey B.G., Pfau M. (2023). Establishing fully-automated fundus-controlled dark adaptometry: A validation and retest-reliability study. Transl. Vis. Sci. Technol..

[42] Simunovic M.P., Mammo Z. (2024). Mechanisms of cone sensitivity loss in retinitis pigmentosa. Ophthalmic. Physiol. Opt..

[43] Pfau M., Lindner M., Fleckenstein M., Finger R.P., Rubin G.S., Harmening W.M., Morales M.U., Holz F.G., Schmitz-Valckenberg S. (2017). Test-retest reliability of scotopic and mesopic fundus-controlled perimetry using a modified MAIA (Macular Integrity Assessment) in normal eyes. Ophthalmologica.

[44] Pfau M., Jolly J.K., Wu Z., Denniss J., Lad E.M., Guymer R.H., Fleckenstein M., Holz F.G., Schmitz-Valckenberg S. (2021). Fundus-controlled perimetry (microperimetry): Application as outcome measure in clinical trials. Prog. Retin. Eye Res..

[45] Aleman T.S., Cideciyan A.V., Windsor E.A.M., Schwartz S.B., Swider M., Chico J.D., Sumaroka A., Pantelyat A.Y., Duncan K.G., Gardner L.M. (2007). Macular pigment and lutein supplementation in ABCA4-associated retinal degenerations. Investig. Ophthalmol. Vis. Sci..

[46] Duncan J.L., Aleman T.S., Gardner L.M., De Castro E., Marks D.A., Emmons J.M., Bieber M.L., Steinberg J.D., Bennett J., Stone E.M. (2002). Macular pigment and lutein supplementation in choroideremia. Exp. Eye Res..

[47] Chew E.Y., Clemons T.E., Jaffe G.J., Johnson C.A., Farsiu S., Lad E.M., Guymer R., Rosenfeld P., Hubschman J.P., Constable I. (2019). Effect of ciliary neurotrophic factor on retinal neurodegeneration in patients with macular telangiectasia type 2: A randomized clinical trial. Ophthalmology.

[48] Owsley C., Jackson G.R., Cideciyan A.V., Huang Y., Fine S.L., Ho A.C., Maguire M.G., Lolley V., Jacobson S.G. (2000). Psychophysical evidence for rod vulnerability in age-related macular degeneration. Investig. Ophthalmol. Vis. Sci..

[49] Ferris F.L., Kassoff A., Bresnick G.H., Bailey I. (1982). New visual acuity charts for clinical research. Am. J. Ophthalmol..

[50] Beck R.W., Moke P.S., Turpin A.H., Ferris F.L., SanGiovanni J.P., Johnson C.A., Birch E.E., Chandler D.L., Cox T.A., Blair R.C. (2003). A computerized method of visual acuity testing: Adaptation of the early treatment of diabetic retinopathy study testing protocol. Am. J. Ophthalmol..

[51] Jolly J.K., Juenemann K., Boagey H., Nadsady M., Bridge H., Maclaren R.E. (2020). Validation of electronic visual acuity (EVA) measurement against standardised ETDRS charts in patients with visual field loss from inherited retinal degenerations. Br. J. Ophthalmol..

[52] Bach M. (1996). The Freiburg visual acuity test - automatic measurement of visual acuity. Optom. Vis. Sci..

[53] Liang J., Williams D.R., Miller D.T. (1997). Supernormal vision and high-resolution retinal imaging through adaptive optics. J. Opt. Soc. Am. A.

[54] Wynne N., Carroll J., Duncan J.L. (2021). Promises and pitfalls of evaluating photoreceptor-based retinal disease with adaptive optics scanning light ophthalmoscopy (AOSLO). Prog. Retin. Eye Res..

[55] Carroll J., Neitz M., Hofer H., Neitz J., Williams D.R. (2004). Functional photoreceptor loss revealed with adaptive optics: An alternate cause of color blindness. Proc. Natl. Acad. Sci. USA.

[56] Scoles D., Sulai Y.N., Langlo C.S., Fishman G.A., Curcio C.A., Carroll J., Dubra A. (2014). In vivo imaging of human cone photoreceptor inner segments. Investig. Ophthalmol. Vis. Sci..

[57] Cideciyan A.V., Jacobson S.G. (2019). Leber congenital amaurosis (LCA): Potential for improvement of vision. Investig. Ophthalmol. Vis. Sci..

[58] Cideciyan A.V. (2010). Leber congenital amaurosis due to RPE65 mutations and its treatment with gene therapy. Prog. Retin. Eye Res..

[59] Jacobson S.G., Cideciyan A.V., Ho A.C., Roman A.J., Wu V., Garafalo A.V., Sumaroka A., Krishnan A.K., Swider M., Mascio A.A. (2022). Night vision restored in days after decades of congenital blindness. iScience.

[60] Russell S.R., Drack A.V., Cideciyan A.V., Jacobson S.G., Leroy B.P., Van Cauwenbergh C., Ho A.C., Dumitrescu A.V., Han I.C., Martin M. (2022). Intravitreal antisense oligonucleotide sepofarsen in Leber congenital amaurosis type 10: A phase 1b/2 trial. Nat. Med..

[61] Cideciyan A.V., Jacobson S.G., Ho A.C., Swider M., Sumaroka A., Roman A.J., Wu V., Russell R.C., Viarbitskaya I., Garafalo A.V. (2023). Durable vision improvement after a single intravitreal treatment with antisense oligonucleotide in CEP290-LCA: Replication in two eyes. Am. J. Ophthalmol. Case Rep..

[62] Pierce E.A., Aleman T.S., Jayasundera K.T., Ashimatey B.S., Kim K., Rashid A., Jaskolka M.C., Myers R.L., Lam B.L., Bailey S.T. (2024). Gene editing for CEP290-associated retinal degeneration. N. Engl. J. Med..

[63] Fischer M.D., Michalakis S., Wilhelm B., Zobor D., Muehlfriedel R., Kohl S., Weisschuh N., Ochakovski G.A., Klein R., Schoen C. (2020). Safety and vision outcomes of subretinal gene therapy targeting cone photoreceptors in achromatopsia: A nonrandomized controlled trial. JAMA Ophthalmol..

[64] Farahbakhsh M., Anderson E.J., Maimon-Mor R.O., Rider A., Greenwood J.A., Hirji N., Zaman S., Jones P.R., Schwarzkopf D.S., Rees G. (2022). A demonstration of cone function plasticity after gene therapy in achromatopsia. Brain.

[65] Michaelides M., Hirji N., Wong S.C., Besirli C.G., Zaman S., Kumaran N., Georgiadis A., Smith A.J., Ripamonti C., Gottlob I. (2023). First-in-human gene therapy trial of AAV8-hCARp.hCNGB3 in adults and children with CNGB3-associated achromatopsia. Am. J. Ophthalmol..

[66] McKyton A., Marks Ohana D., Nahmany E., Banin E., Levin N. (2023). Seeing color following gene augmentation therapy in achromatopsia. Curr. Biol..

[67] Anderson E.J., Dekker T.M., Farahbakhsh M., Hirji N., Schwarzkopf D.S., Michaelides M., Rees G. (2024). fMRI and gene therapy in adults with CNGB3 mutation. Brain Res. Bull..

[68] Zein W.M., Jeffrey B.G., Wiley H.E., Turriff A.E., Tumminia S.J., Tao W., Bush R.A., Marangoni D., Wen R., Wei L.L. (2014). CNGB3-achromatopsia clinical trial with CNTF: Diminished rod pathway responses with no evidence of improvement in cone function. Investig. Ophthalmol. Vis. Sci..

[69] Cideciyan A.V., Swider M., Aleman T.S., Roman M.I., Sumaroka A., Schwartz S.B., Stone E.M., Jacobson S.G. (2007). Reduced-illuminance autofluorescence imaging in ABCA4-associated retinal degenerations. J. Opt. Soc. Am. A.

[70] Cideciyan A.V., Jacobson S.G., Ho A.C., Krishnan A.K., Roman A.J., Garafalo A.V., Wu V., Swider M., Sumaroka A., Van Cauwenbergh C. (2022). Restoration of cone sensitivity to individuals with congenital photoreceptor blindness within the phase 1/2 sepofarsen trial. Ophthalmol. Sci..

[71] Li R.T.H., Roman A.J., Sumaroka A., Stanton C.M., Swider M., Garafalo A.V., Heon E., Vincent A., Wright A.F., Megaw R. (2023). Treatment strategy with gene editing for late-onset retinal degeneration caused by a founder variant in C1QTNF5. Investig. Ophthalmol. Vis. Sci..

[72] Jacobson S.G., Voigt W.J., Parel J.M., Apathy P.P., Nghiem-Phu L., Myers S.W., Patella V.M. (1986). Automated light- and dark-adapted perimetry for evaluating retinitis pigmentosa. Ophthalmology.

[73] Cideciyan A.V., Pugh E.N., Lamb T.D., Huang Y., Jacobson S.G. (1997). Rod plateaux during dark adaptation in Sorsby’s fundus dystrophy and vitamin A deficiency. Investig. Ophthalmol. Vis. Sci..

[74] Cideciyan A.V., Zhao X., Nielsen L., Khani S.C., Jacobson S.G., Palczewski K. (1998). Null mutation in the rhodopsin kinase gene slows recovery kinetics of rod and cone phototransduction in man. Proc. Natl. Acad. Sci. USA.

[75] Cideciyan A.V., Haeseleer F., Fariss R.N., Aleman T.S., Jang G.F., Verlinde C.L.M.J., Marmor M.F., Jacobson S.G., Palczewski K. (2000). Rod and cone visual cycle consequences of a null mutation in the 11-cis-retinol dehydrogenase gene in man. Vis. Neurosci..

[76] Stockman A., Sharpe L.T., Fach C. (1999). The spectral sensitivity of the human short-wavelength sensitive cones derived from thresholds and color matches. Vision Res..

[77] Stockman A., Sharpe L.T. (2000). The spectral sensitivities of the middle- and long-wavelength-sensitive cones derived from measurements in observers of known genotype. Vision Res..

[78] Barbur J.L., Rodriguez-Carmona M., Evans B.E.W. (2021). Color vision assessment-3. An efficient, two-step, color assessment protocol. Color Res. Appl..

[79] Barbur J.L., Rodriguez-Carmona M. (2017). Colour vision requirements in visually demanding occupations. Br. Med. Bull..

[80] Barbur J.L., Harlow A.J., Plant G.T. (1994). Insights into the different exploits of colour in the visual cortex. Proc. Biol. Sci..

[81] Dubra A., Sulai Y. (2011). Reflective afocal broadband adaptive optics scanning ophthalmoscope. Biomed. Opt. Express.

[82] Morgan J.I.W., Jiang Y.Y., Vergilio G.K., Serrano L.W., Pearson D.J., Bennett J., Maguire A.M., Aleman T.S. (2022). Short-term assessment of subfoveal injection of adeno-associated virus-mediated hCHM gene augmentation in choroideremia using adaptive optics ophthalmoscopy. JAMA Ophthalmol..

[83] Dubra A., Harvey Z. (2010). Registration of 2D images from fast scanning ophthalmic instruments. Lecture Notes Comput. Sci..

[84] Chen M., Cooper R.F., Han G.K., Gee J., Brainard D.H., Morgan J.I. (2016). Multi-modal automatic montaging of adaptive optics retinal images. Biomed. Opt. Express.

