# Supplementary material for: Evaluation of Retinal Structure and Visual Function in Blue Cone Monochromacy to Develop Clinical Endpoints for L-opsin Gene Therapy
Source: Int J Mol Sci. 2024 Oct 2;25(19):10639. doi: 10.3390/ijms251910639 (PMC11477341; doi:10.3390/ijms251910639)

Supplementary Materials for

Evaluation of Retinal Structure and Visual Function in Blue Cone Monochromacy to Develop Clinical Endpoints for L-opsin Gene Therapy

Artur V. Cideciyan [1,*], Alejandro J. Roman [1], Raymond L. Warner [1], Alexander Sumaroka [1], Vivian Wu [1], Yu Y. Jiang [1], Malgorzata Swider [1], Alexandra V. Garafalo [1], Iryna Viarbitskaya [1], Robert C. Russell [1], Susanne Kohl [2], Bernd Wissinger [2], Caterina Ripamonti [3], John L. Barbur [4], Michael Bach [5], Joseph Carroll [6], Jessica I. W. Morgan [1] and Tomas S. Aleman [1]

Supplementary Figure S1: Comparison of temporal vs spatial averaging on outer retinal subcellular defects. Comparison of temporally average Spectralis OCT scans (left column) and spatially averaged Optovue OCT scans (right column). Yellow arrows highlight defects at the IS/OS layer that are visible on spatially averaged images.

Supplementary Figure S2: Setup parameters used in the public domain web-based FrACT10 application to measure chromatic acuities with diagonal gratings.

Supplementary Figure S3: Setup parameters used in the commercial CAD application to measure color vision.

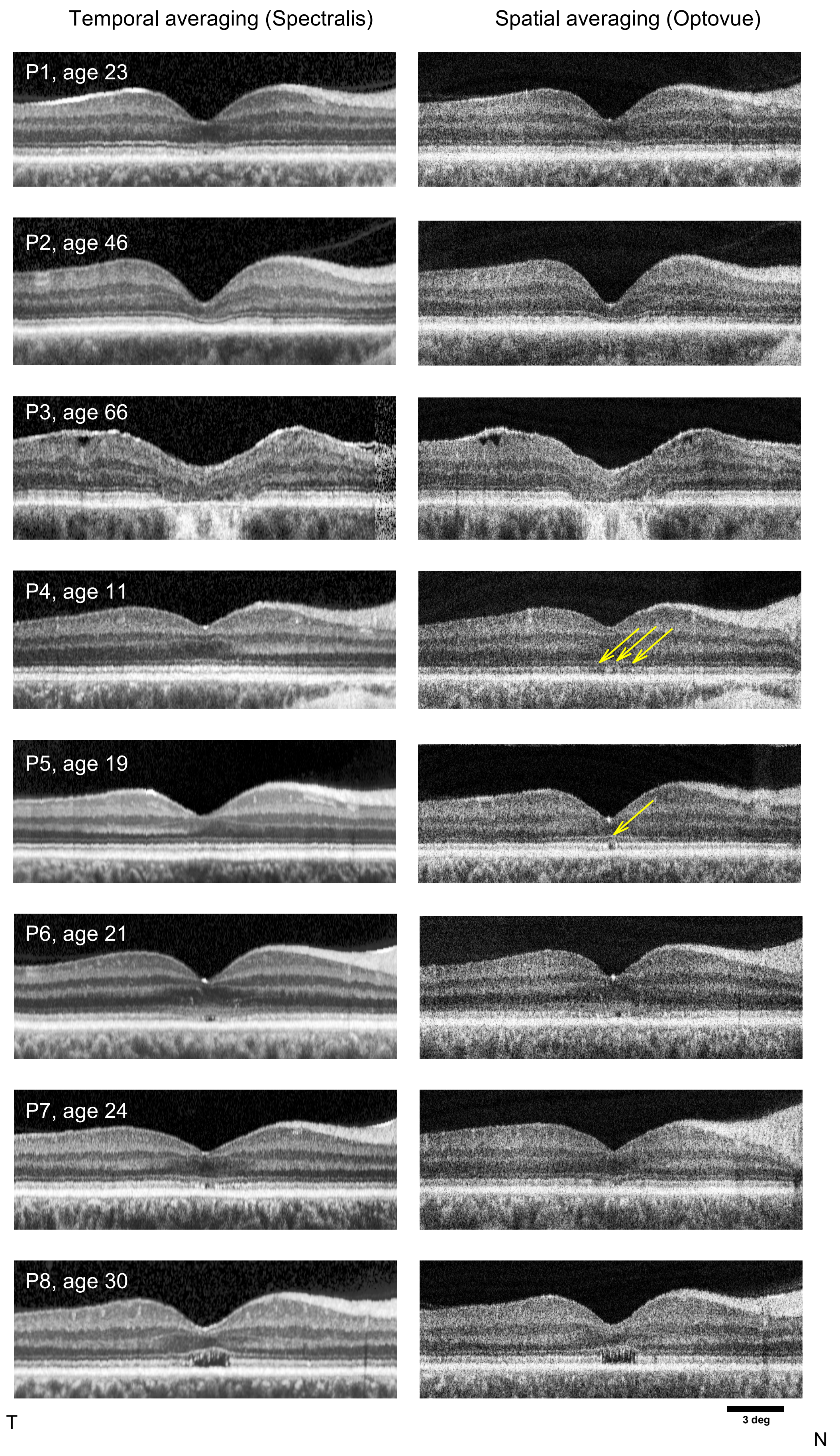
Temporal averaging (Spectralis)
Spatial averaging (Optovue)
P1, age 23
P2, age 46
P3, age 66
P4, age 11
P5, age 19
P6, age 21
P7, age 24
P8, age 30
T
3 deg
N

Supplementary Figure S2

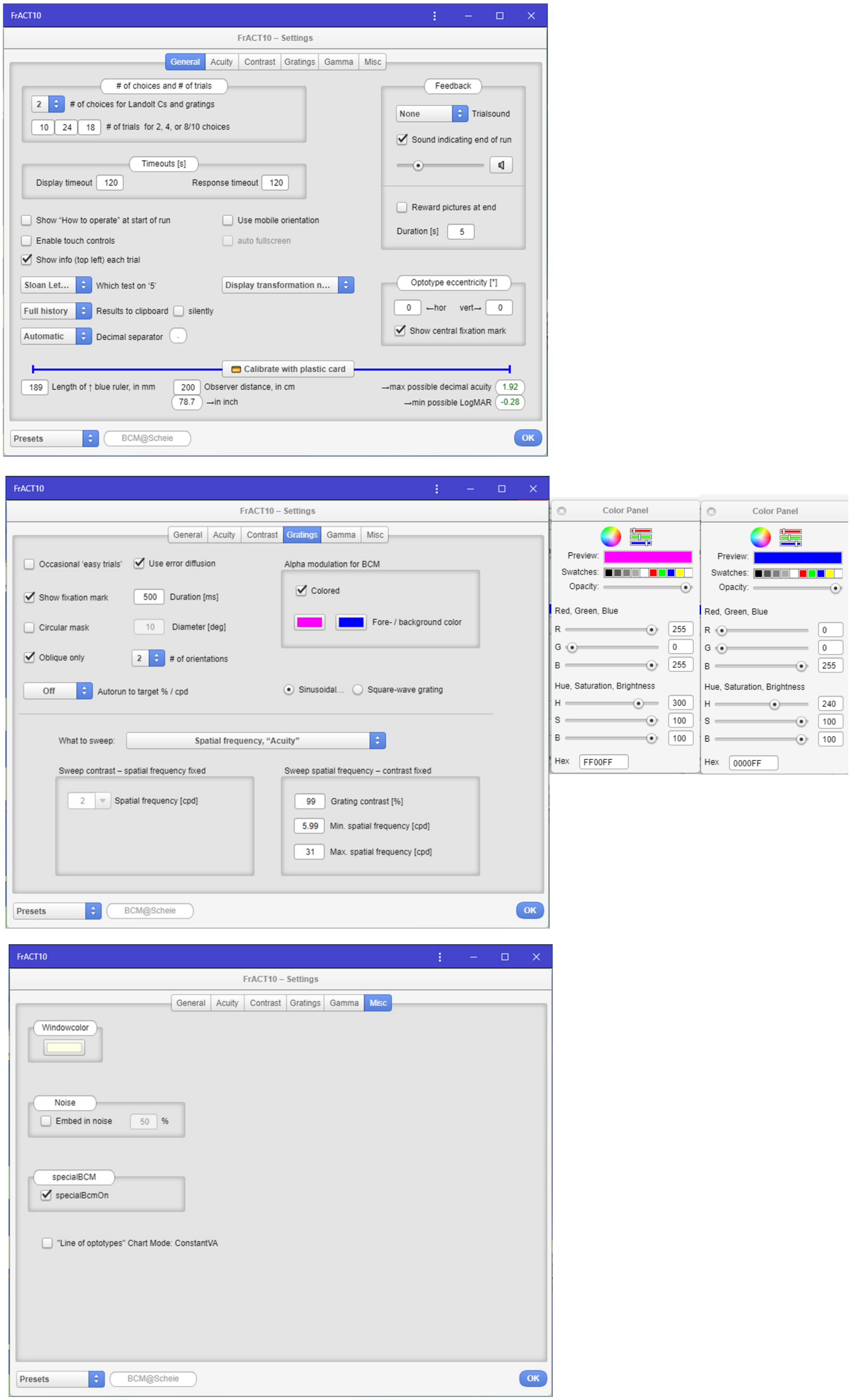

Supplementary Figure S3

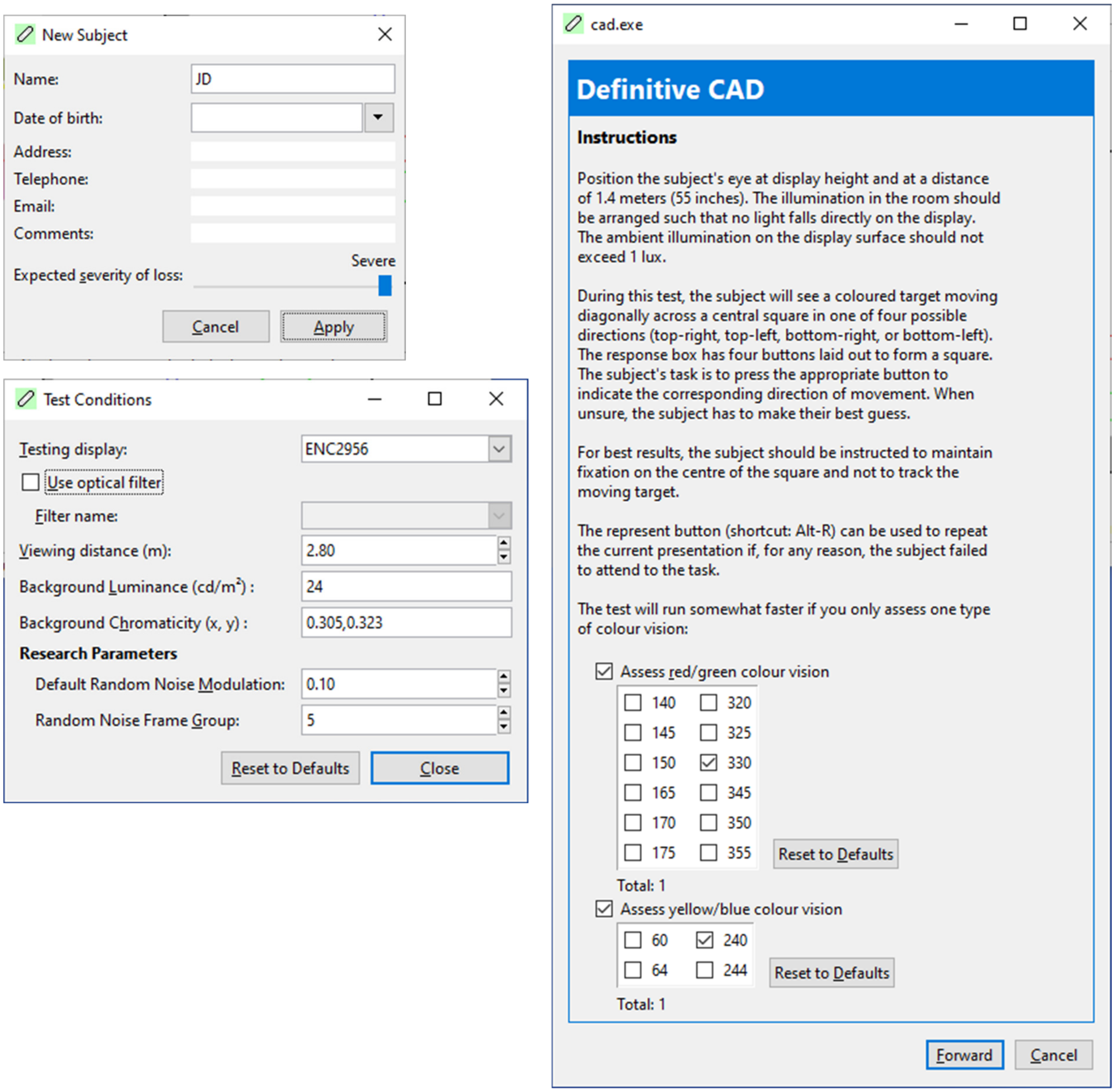

Supplement: Supplementary file 1 [file ijms-25-10639-s001.zip › ijms-3223761-supplementary.pdf]
